# Affective Sensitivity to Air Pollution (ASAP): Person-specific associations between daily air pollution and affective states

**DOI:** 10.1371/journal.pone.0307430

**Published:** 2024-08-07

**Authors:** Michelle Ng, Denis Gerstorf, David E. Conroy, Aaron L. Pincus, Nilàm Ram

**Affiliations:** 1 Department of Communication, Stanford University, Palo Alto, California, United States of America; 2 Emmett Interdisciplinary Program in Environment and Resources, Stanford University, Palo Alto, California, United States of America; 3 Department of Psychology, Humboldt University of Berlin, Berlin, Germany; 4 German Socio-Economic Panel (SOEP) at German Institute for Economic Research (DIW), Berlin, Germany; 5 Department of Kinesiology, The Pennsylvania State University, University Park, Pennsylvania, United States of America; 6 Department of Psychology, Pennsylvania State University, University Park, Pennsylvania, United States of America; 7 Department of Psychology, Stanford University, Palo Alto, California, United States of America; Griffith University, AUSTRALIA

## Abstract

Individuals’ sensitivity to climate hazards is a central component of their vulnerability to climate change. In this paper, we introduce and outline the utility of a new intraindividual variability construct, *affective sensitivity to air pollution* (ASAP)–defined as the extent to which an individual’s affective states fluctuate in accordance with daily changes in air quality. As such, ASAP pushes beyond examination of differences in individuals’ *exposures* to air pollution to examination of differences in individuals’ *sensitivities* to air pollution. Building on known associations between air pollution exposure and adverse mental health outcomes, we empirically illustrate how application of Bayesian multilevel models to intensive repeated measures data obtained in an experience sampling study (*N* = 150) over one year can be used to examine whether and how individuals’ daily affective states fluctuate with the daily concentrations of outdoor air pollution in their county. Results indicate construct viability, as we found substantial interindividual differences in ASAP for both affect *arousal* and affect *valence*. This suggests that repeated measures of individuals’ day-to-day affect provides a new way of measuring their sensitivity to climate change. In addition to contributing to discourse around climate vulnerability, the intraindividual variability construct and methodology proposed here can help better integrate affect and mental health in climate adaptation policies, plans, and programs.

## Introduction

Over the past two decades, conceptual thinking around vulnerability to climate change has undergone a paradigm shift [1–3]. In the early 2000s, the Intergovernmental Panel on Climate Change (IPCC) defined climate vulnerability as a function of one’s *exposure*, *sensitivity*, and *adaptive capacity* to climate change [4]. Under this older paradigm, where *exposure* and *sensitivity* to climate change were treated as “almost inseparable properties of a system” [5], measures of *exposure* were often used as proxy indicators of *sensitivity*. The two constructs were viewed as so coupled that measuring one was good enough. More recently, however, the IPCC decoupled *sensitivity* from *exposure*. Climate vulnerability was redefined as a function of one’s *sensitivity* and *adaptive capacity* to climate change [6, 7]–existing within the context of, but not identical to, one’s *exposure*. Under this newer paradigm, *sensitivity* to climate change should no longer be proxied by *exposure* to climate change [3]. The new approach raises the question: How might we measure sensitivity to climate change?

Following the IPCC’s conceptual shift in defining climate vulnerability, we propose that intensive longitudinal data on individuals’ *daily affect* obtained in experience sampling studies can be used to measure sensitivity to climate change. Recognizing that climate vulnerability is largely produced by power relations and other socio-political processes [8, 9], we view psychological sensitivity to climate change as one indicator within a larger, complex system of individuals’ and communities’ overall sensitivity to climate change. We focus on affect as a measure of sensitivity for three reasons. First, affect is a reliable, sensitive, and time-varying marker of individuals’ responses to their environments [10]. Individuals’ affective states change from moment to moment and from day to day—continually providing information about how they are doing, whether there are threats in their environments, and whether they should approach or avoid those threats (e.g., anger, sadness). Studying day-to-day fluctuations in individuals’ affective states reveals if and how individuals are influenced by changes in their everyday environments. For example, systematic increases in individuals’ daily negative affect that are coupled with daily stressors indicate the extent to which individuals are reactive to those stressors [11]. There are also interindividual differences in affective reactivity, such that different individuals respond differently to changes in their environment [12]. For instance, individuals who are higher in neuroticism tend to have greater reactivity to daily stressors [13–15]; and older adults may experience less pronounced fluctuations in their affect compared to younger adults [16, 17]. Examining how individuals’ affect fluctuates in accordance with changes in air pollution can illuminate whether and how air pollution exposure is experienced differently across individuals—thus providing a new way to measure sensitivity to climate change.

Second, affect is a defining characteristic of aspects of mental health that are associated with both climate change and air pollution exposure. In its most recent assessment report, the IPCC [7] states with “very great confidence” that climate change adversely influences mental health through numerous pathways; and as detailed below, prior research has established linkages between mental health and air pollution exposure specifically. Given that disturbed affect and affect regulation play a role in 40–75% of mental disorders [18–21], it is likely that affect is a mechanism through which air pollution exposures influence mental health. Examining how individuals’ affect fluctuates in accordance with daily changes in air pollution can provide new insight into whether and how day-to-day affective dynamics induced by air pollution contribute to changes in or differences between individuals’ mental health and wellbeing.

Third, individuals’ affect is a key driver of their climate change mitigation behaviors, such as recycling and conserving energy, and adaptation behaviors, such as purchasing insurance and evacuating. Various theories, such as the Extended Parallel Process Model and Climate Change Risk Perception Model, postulate that affect informs behavioral decision-making [22, 23]. Accordingly, empirical evidence shows that negative affect is the largest predictor of individuals’ climate risk perception [23] and willingness to engage in climate mitigation behaviors [24], as well as one of the largest predictors of individuals’ climate adaptation behaviors [25, 26]. Put differently, affect is a cornerstone of climate action. If air pollution blunts individuals’ affect in some way, individuals may perform fewer mitigation and adaptation behaviors—with implications for climate change and their personal wellbeing [27]. Examining how an individual’s affect fluctuates in accordance with daily changes in air pollution thus illuminates another pathway by which an individual’s exposure to climate hazards may indirectly influence their climate actions.

### Air pollution and wellbeing

The intensification of air pollution due to climate change (e.g., particulate matter from wildfire smoke, ozone from hotter temperatures) is making air an increasingly visible part of many individuals’ daily experiences [28–31]. Already 90% of the population worldwide, especially communities of color and low-income communities, breathes air that does not meet the World Health Organization’s guidelines for livable air quality [32–34]. This is dangerous because exposure to carbon monoxide (CO), nitrogen oxides (NO_x_), ozone (O_3_), sulfur dioxide (SO_2_), and particulate matter (PM_2.5_ and PM_10_) is associated with a variety of health problems, including premature mortality [35], cardiovascular disease and serious cardiac events [36], asthma attacks [37], and lung cancer [38].

Exposure to air pollution is also associated with a variety of psychological states and disorders, including higher levels of perceived stress in older men [39], higher stress responsivity among adolescent girls at risk for anxiety disorders [40], increased odds of depressive and anxiety symptoms in older adults [41], increased risk of emergency department visits for youth with diagnosed mental illnesses [42], and the development of psychopathology in 18-year-olds who had been exposed to air pollution as children and adolescents [43]. The links between mental health and long-term exposure to air pollution suggest that fluctuations in *affect*—a core element of mental health—may reflect the short-term effects of air pollution on individuals’ wellbeing. In addition, individuals’ affect to be sensitive to daily changes in air pollution because affect-driving health behaviors, such as physical activity and high-quality sleep, are compromised by air pollution [44–50]. Given these links, the extent to which an individual’s daily affective states fluctuate in accordance with their daily exposures to air pollution should provide a new way to measure sensitivity to climate change.

### Affective Sensitivity to Air Pollution (ASAP)

**Fig 1:**
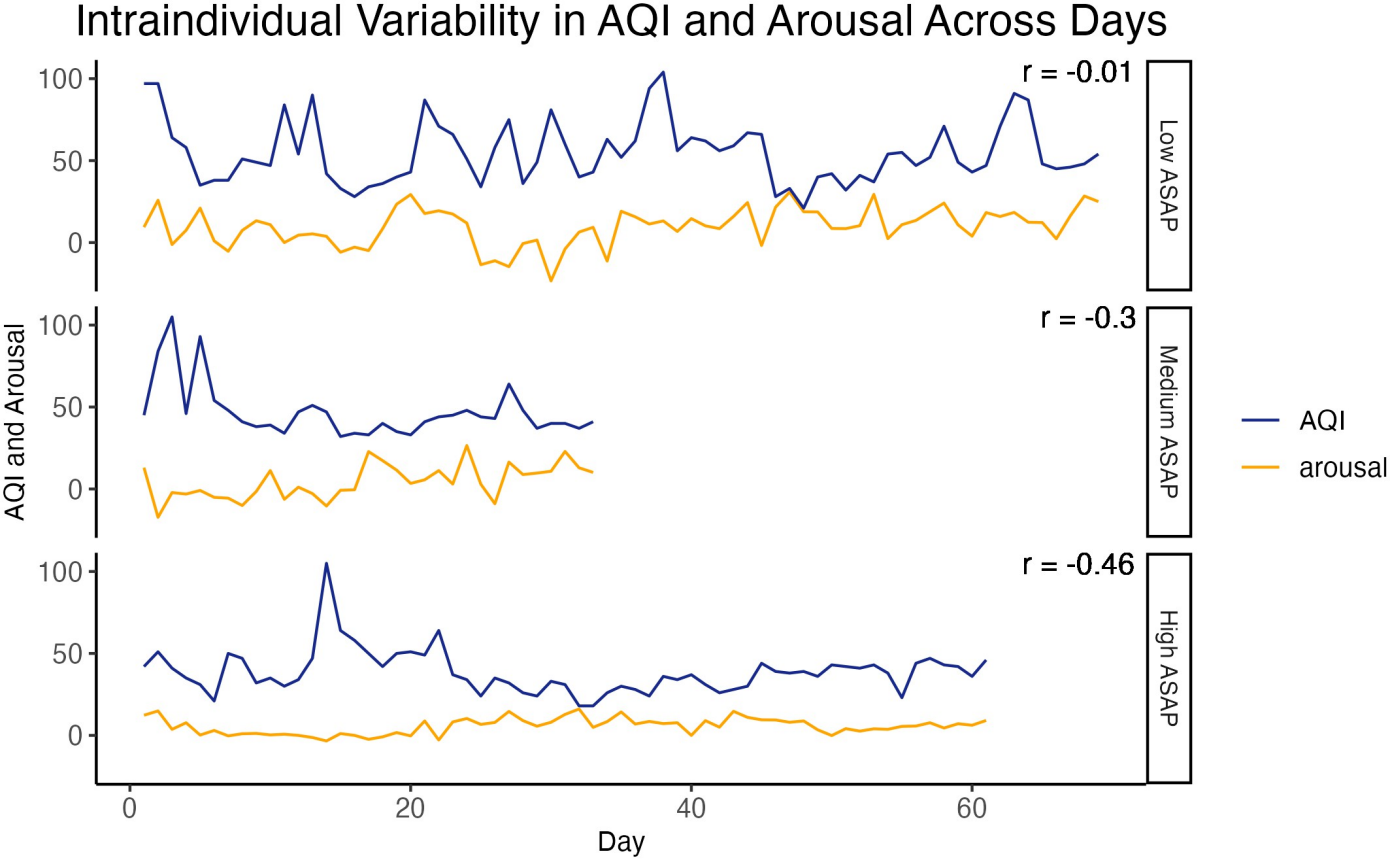
Intraindividual variability in air pollution and affect *arousal* for three individuals with different levels of affective sensitivity to air pollution (ASAP). Fluctuations in air pollution (blue line) and affect arousal (orange line) over time (x-axis) are shown for three individuals (rows). The correlation coefficient, *r*, indicates the strength of covariation for each individual’s air pollution and affect arousal.

Building from prior research on individuals’ psychological sensitivity to changes in their everyday environments and life experiences [15, 17, 51, 52], we propose using a new intraindividual variability construct, *affective sensitivity to air pollution* (ASAP), to measure psychological sensitivity to climate change. Formally, ASAP is defined as the extent to which an individual’s affective states fluctuate in accordance with daily changes in air pollution. ASAP is measured using experience sampling methodologies, wherein the reports individuals provide every few hours about their psychological states (e.g., ecological momentary assessments) are combined with environmental data obtained from local air quality monitors. As illustrated in Fig 1, individuals with higher ASAP experience greater fluctuations in their affective states in accordance with daily changes in air pollution compared to individuals with lower ASAP.

In this paper, we explore whether and how individuals’ daily affective states fluctuate in accordance with their daily exposures to air pollution. Our goals are twofold: (1) to introduce the concept of *affective sensitivity to air pollution* (ASAP) and (2) to illustrate how ASAP can be measured using available empirical data. Using Bayesian multilevel models that isolate within-person covariation, we demonstrate how intensive longitudinal data enable examination of the prototypical individual’s ASAP, interindividual differences in ASAP, and whether interindividual differences in ASAP are related to overall levels of chronic exposure to air pollution. We conclude with a discussion of how measurement of ASAP—as one form of sensitivity to climate change—can inform climate adaptation policies, plans, and programs. While our proposal and empirical illustration focus on individuals’ ASAP, they straightforwardly transfer to assessments of any climate hazard and outcome variable of interest.

## Materials and method

Our empirical illustration of individuals’ ASAP combines two data sources. Data on individuals’ daily affective states were obtained in the Intraindividual Study of Affect, Health, and Interpersonal Behavior (iSAHIB), an intensive longitudinal study that collected rich repeated measures of its namesake variables in everyday life [53]. Data on daily levels of air pollution were obtained from the United States Environmental Protection Agency’s (EPA) compilation of air pollution data from outdoor monitors located across the United States [54]. Data from iSAHIB has previously been used to examine intraindividual associations between physical activity and life satisfaction [55], alcohol use and sleep quality [56], interpersonal behavior and personality [57], and social interactions and core affect [58]. Here, these daily affect data are for the first time combined with environmental (in this case, air pollution) data.

### Participants & procedure

The iSAHIB study protocol (#33706) was approved by the Institutional Review Board of Pennsylvania State University. Written consent was obtained from participants. The iSAHIB sample consisted of 150 adults (51% women) living around central Pennsylvania, USA, specifically in Centre County (*n* = 134), nearby counties in Pennsylvania (*n* = 3 each in Blair, Clearfield, and Clinton Counties; *n* = 1 each in Cumberland, Lehigh, Lycoming, Mifflin, and Montgomery Counties), and nearby counties in Maryland and New Jersey (*n* = 1 each in Carrol and Somerset Counties). Participants were between 18 and 89 years of age (M_age_ = 47.10, SD_Age_ = 18.76) and had obtained between 2 and 24 years of formal education (M_Educ_ = 16.36, SD_Educ_ = 3.90). Half were employed full-time; 64% were engaged, married, or in committed relationship (15% single); 79% lived with family (15% with roommates, etc., 6% alone); 91% self-identified as White (4% African American, 1% Asian American, and 4% multiracial or some other race); and 93% as heterosexual.

After being recruited between April 23, 2010 and April 18, 2011, informed about the intensive assessment protocol, and providing written informed consent, individuals contributed extensive reports about their lives during the next year through a combination of web-based (completed during visits to the laboratory) and smartphone-based (completed multiple times per day during daily life) questionnaires. Most relevant for our illustration of ASAP, participants responded to daily surveys (52 items) on their study-provided smartphone each evening for three 21-day measurement bursts (9 weeks total) over the course of one year (about 4.5-month intervals between bursts) between May 2010 and July 2011. Of the 150 participants, 136 (90.7%) completed the entire intensive protocol, 11 (7.3%) completed one-third of the protocol, and 3 (2.0%) completed two-thirds of the protocol. Participants who withdrew after partial completion did not differ systematically from those who completed the entire protocol with respect to the measured demographics (*p*s >.05). Some participants also completed extra days until they returned the phone and formally exited the study. Overall, participants provided 8,541 daily reports of affect. This analysis makes use of 8,250 (96.6%) of these daily reports that could be linked to daily air pollution data (M = 56.5 daily reports; SD = 13.0; range 13 to 76).

### Measures

#### Daily air pollution

Individuals’ daily exposure to air pollution, specific to the county in which they lived, was measured using the United States’ Air Quality Index (AQI). The AQI is calculated and issued each day by the EPA based on concentrations of the five major pollutants regulated by the Clean Air Act: ground-level ozone, particulate matter, carbon monoxide, sulfur dioxide, and nitrogen dioxide [59]. The AQI value for a given day is the maximum concentration observed across the five pollutants (each scaled such that 100 is the national public health air quality standard for that pollutant). On a scale from 0 to 500, the AQI communicates the daily level of air pollution and its associated health concern: 0 to 50 (“Good”), 51 to 100 (“Moderate”), 101 to 150 (“Unhealthy for Sensitive Groups”), 151 to 200 (“Unhealthy”), 201 to 300 (“Very Unhealthy”), and 301 and higher (“Hazardous).

County-level AQI data for the dates of iSAHIB data collection were downloaded from the EPA’s data repository [54]. Two counties did not have any available AQI data, four counties had AQI data based on one major pollutant, two counties had AQI data based on two major pollutants, two counties had AQI data based on three major pollutants, and one county had AQI data based on four major pollutants. Due to these gaps, AQI was available for 96.6% of the daily reports obtained across iSAHIB (8,250 of 8,541 person-days). Following the available AQI data, we focused our analysis on daily reports that could be paired with AQI: 97.3% of the daily reports obtained in Burst 1 of iSAHIB (2,972 of 3,056 person-days); 95.6% of Burst 2 (2,672 of 2,795 person-days); and 96.9% of Burst 3 (2,606 of 2,690 person-days). Examination of missingness patterns suggested that the missing AQI data could reasonably be treated as missing at random.

#### Daily affect (*arousal* and *valence*)

In all three bursts of the iSAHIB, individuals’ daily affective states were measured at the end of each day by asking participants to indicate how strongly they felt 20 emotions. Participants responded to items such as “Today I felt [HAPPY]” using a slider-type interface with end-point anchors labeled “Not at all…Strongly” that was digitally coded on a 0 to 100 scale (numbers not visible to participants). Even distribution of the 20 emotion-specific items across all four quadrants of the core affect circumplex [60] facilitated calculation of daily affect *arousal*, which describes the level of physiological activation involved in an individual’s affective state, and daily affect *valence*, a hedonistic evaluation describing the positivity or negativity of an individual’s affective state. Specifically, following Kuppens et al. [61], *arousal* on day *t* for person *i* was calculated as,

$$AffectArousal_{ti}=\left({\text{HAP}}_{ti}+{\text{HAN}}_{ti}\right)-\left({\text{LAP}}_{ti}+{\text{LAN}}_{ti}\right)$$ (1a)

and *valence* on day *t* for person *i* was calculated as,

$$AffectValence_{ti}=\left({\text{HAP}}_{ti}+{\text{LAP}}_{ti}\right)-\left({\text{HAN}}_{ti}+{\text{LAN}}_{ti}\right)$$ (1b)

where *HAP*_*ti*_ is the mean of person *i*’s reports on day *t* for the five high arousal positive emotions: alert, happy, enthusiastic, excited, and proud; *HAN*_*ti*_ is the mean of person *i*’s reports on day *t* for the five high arousal negative emotions: embarrassed, nervous, stressed, tense, and upset; *LAP*_*ti*_ is the mean of person *i*’s reports on day *t* for the five low arousal positive emotions: calm, content, peaceful, relaxed, and satisfied; and *LAN*_*ti*_ is the mean of person *i*’s reports on day *t* for the five low arousal negative emotions: bored, depressed, disappointed, sad, and sluggish. For convenience of interpretation, resulting scores for both *arousal* and *valence* were divided by 2 so they range from -100 to 100.

#### Day

To control for systematic linear time trends in daily affect that may have occurred over the course of the 9-week study period, *Day* in study was included as a covariate.

### Data configuration and analysis

As outlined above, ASAP is defined as the extent to which fluctuations in an individual’s affect are related to fluctuations in air pollution levels—that is, the *intraindividual covariation* of daily air pollution (AQI) scores and daily affect scores. Illustrative plots of three individuals’ daily *AQI* and affect *arousal* are shown in Fig 1. For the participant represented in the top panel, fluctuations in *AQI* and levels of *arousal* do not appear associated; while for the participants represented in the middle and bottom panels, fluctuations in *AQI* and levels of *arousal* are systematically linked in that higher levels of AQI tend to coincide with lower levels of *arousal*. Interindividual differences in the strength of ASAP indicated by differences in the extent of covariation (e.g., *r* = -0.01 versus *r* = -0.30 versus *r* = -0.46).

Formally, individuals’ ASAP and interindividual differences in ASAP were examined in a Bayesian multilevel modeling framework [62] that accommodated the nested nature of the intensive longitudinal data (daily repeated measures nested within persons) and enabled robust inference. Following usual practice in analysis of intensive longitudinal data [63], the daily AQI reports were separated into within-person and between-person components prior to analysis. Specifically, *overall air pollution*_*i*_ scores were calculated for each participant as the within-person mean of all their repeated measures of AQI. *Daily air pollution*_*ti*_ scores were then calculated as that day’s deviation from the individual’s *overall air pollution*_*i*_ score. The within- and between-person associations of daily affect and air pollution were then examined using 2-level models of the form,

$$Affect_{ti}=\beta_{0\text{i}}+\beta_{1\text{i}}\left(Daily\;air\;pollution_{ti}\right)+\beta_{2\text{i}}(Day_{ti})+e_{ti}$$ (2)

where the repeated measures of daily affect (valence and arousal examined in separate models) for day *t* for individual *i*, *Affect*_*ti*_, is modeled as a function of person-specific intercepts, β_0i_, that indicate an individual’s baseline level of affect; person-specific slope coefficients, β_1i_, that indicate an individual’s ASAP; person-specific time-related trends, β_2i_; and residual error, e_ti_, that is assumed normally distributed with standard deviation σ_e_. Person-specific coefficients were simultaneously modeled as a function of the person-level exposure variables,

$$\beta_{0\text{i}}=\gamma_{00}+\;\gamma_{01}(Overall\;air\;pollution_{i})\;+u_{0i}$$ (3)

$$\beta_{1\text{i}}=\gamma_{10}+\;\gamma_{11}(Overall\;air\;pollution_{i})+u_{1i}$$ (4)

$$\beta_{2\text{i}}=\gamma_{20}$$ (5)

where the *γ*s are sample-level parameters that describe the prototypical levels of baseline affect and ASAP and how between-person differences in baseline affect and ASAP are associated with differences in overall level of exposure to air pollution, and the *u*s are residual unexplained between-person differences that are assumed multivariate normally distributed with standard deviations σ_u0_ and σ_u1_, and correlation *r*_u0u1_. Person-level predictors were sample-mean centered. In line with our treatment of *Day*_*ti*_ as a nuisance covariate, random effects for the linear time trends were not included. Model explorations indicated no substantial differences in the pattern of findings across a variety of alternative random effects structures.

We expect that for the prototypical person, days with higher air pollution exposure will be accompanied by changes in affect *arousal* and affect *valence* (γ_10_). Previously noted associations between air pollution exposure and depressive symptoms suggest that on days when air pollution is higher, affect *arousal* will be lower and affect *valence* will be lower. However, previously noted associations between air pollution exposure and anxiety symptoms suggest that on days when air pollution is higher, affect *arousal* will be higher. Similarly mixed findings around habituation versus sensitization suggest that chronic exposure to air pollution will impact ASAP (γ_11_), but the direction of impact is unclear. When facing chronic exposure to any stimulus, individuals may exhibit greater affective sensitivity (sensitization) or reduced affective sensitivity (habituation) [52]. Past research has suggested that affect regulation skills may be acquired over multiple exposures to a stimulus, as individuals learn how to adapt [17, 64]. Following this logic, we can expect that, over the long-term, individuals become more or less sensitive to changes in air pollution through sensitization or habituation processes, such that differences in overall exposure moderate extent of ASAP. Overall, our analysis extends prior research linking air pollution exposures and adverse mental health outcomes by examining individuals’ ASAP at a daily time-scale.

The multilevel models were estimated in a Bayesian analysis framework using the *brms* package in R [65]. Estimation was based on two chains of 2,000 iterations (500 of which were warm-up) that provided a total of 3,000 samples for the posterior distributions. We used the mildly informative default priors, a half Student’s-t prior with 3 degrees of freedom and scale parameters between 8.9 to 26.7 (depending upon outcome variable) for standard deviations of random effects, a Lewandowski—Kurowicka—Joe prior with parameter of 1 for correlations among random effects, and flat or Student’s-t priors with mildly informative means for regression coefficients. Incomplete data were treated using standard missing at random assumptions. Convergence of the Markov Chain Monte Carlo (MCMC) algorithms was determined through graphical checks of the chains and posterior distributions, inspection of R-hat values, and posterior predictive checks, all of which suggested that MCMC chains had converged. In follow-up sensitivity analyses, we examined subsets of the emotions (i.e., positive high arousal emotions, positive low arousal emotions, negative high arousal emotions, and negative low arousal emotions). The same general pattern of results reported in the main analysis also manifested in the sensitivity analyses (see S1 Table). Following the Bayesian estimation, substantive inferences are obtained from examination of 95% credible intervals and probability of direction (which is interpreted similarly to a *p*-value) obtained from the posterior distributions of each model parameter.

## Results

We examined the strength of ASAP for different individuals, the direction of ASAP, and whether interindividual differences in ASAP are related to overall exposure to air pollution. Separate models were used to examine sensitivity of affect *arousal* and affect *valence*. Descriptive statistics are included in Table 1.

**Table 1:** Descriptive statistics for air pollution and two characteristics of affective states: *Arousal* and *valence*.

	Between-person						Within-person			
	M_*B*_	SE_B_	SD_*B*_	Air pollution_*B*_	Arousal_*B*_	Valence_*B*_	SD_*w*_	Air pollution_*w*_	Arousal_*w*_	Valence_*w*_
Air pollution (0 to 500)	50.13	0.47	5.64	1.00			17.37	1.00		
Arousal (-100 to 100)	0.14	0.60	7.20	–0.08	1.00		8.59	–0.03	1.00	
Valence (-100 to 100)	36.03	1.78	21.49	0.04	–0.02	1.00	18.17	0.01	–0.20	1.00

Analysis is based on 8,250 days nested within 150 persons. *B* and *W* subscripts indicate person-level and within-person (i.e., day-level) variables, respectively. *M*_*B*_ = mean of the person-level variable; SE_B_ = standard error of *M*_*B*_; *SD*_*B*_ = standard deviation of the person-level variable. *SD*_*w*_ = average within-person standard deviation of the day-level variable.

**Fig 2:**
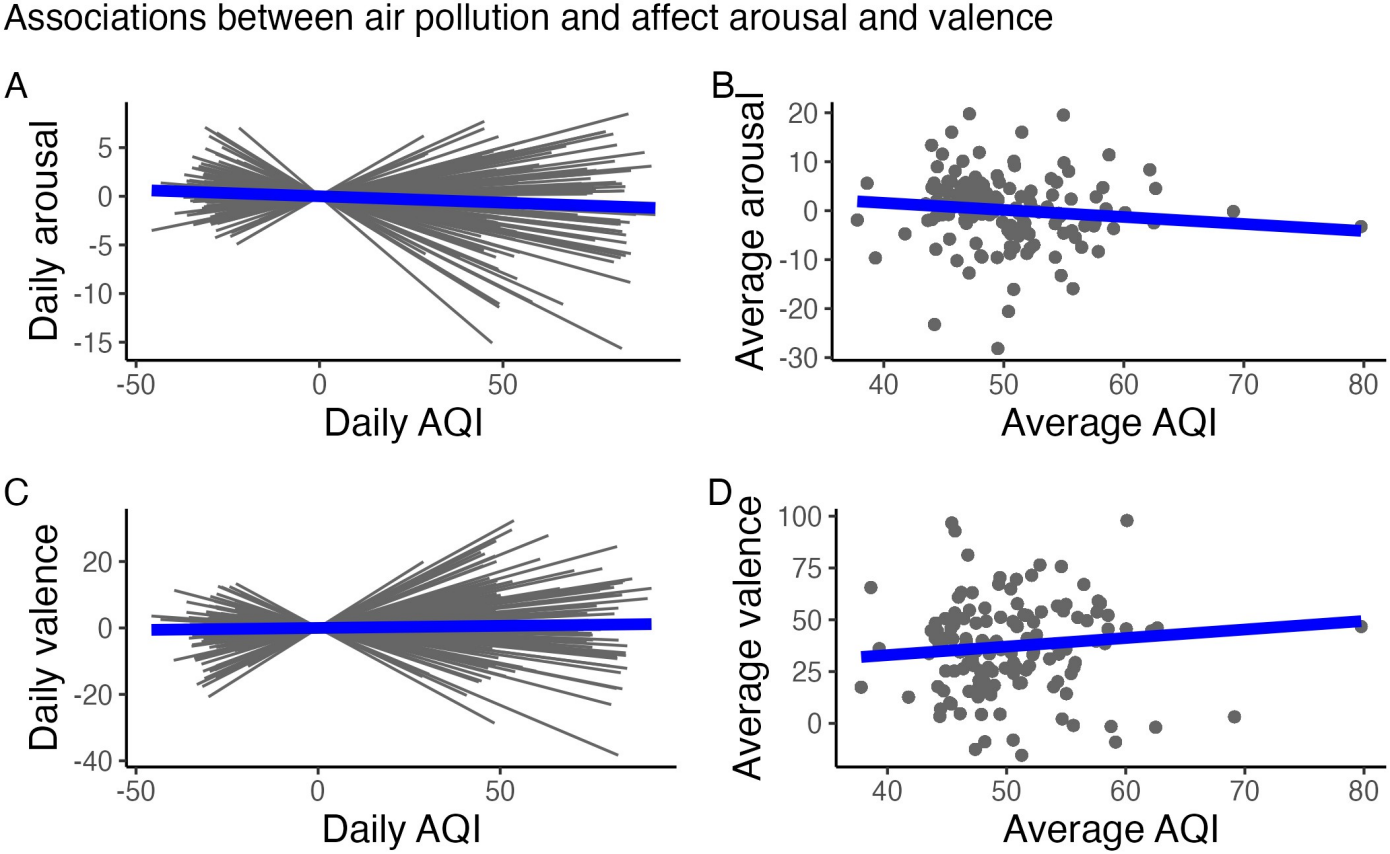
Within-person and between-person associations for affect and air pollution. Panel A: Within-person associations of daily air pollution and daily *arousal*. Panel B: Between-person associations of overall air pollution and overall *arousal*. Panel C: Within-person associations of daily air pollution and daily *valence*. Panel D: Between-person associations of overall air pollution and overall *valence*. In Panels A and C, the thin black lines show interindividual differences in affective sensitivity to air pollution (ASAP), while the thick blue line represents the prototypical individual’s ASAP.

Results for models examining how daily air pollution is associated with fluctuations in affect *arousal* and affect *valence* are included in Table 2 and illustrated in Fig 2.

**Table 2:** Results from multilevel models for affect *arousal* and affect *valence*.

		Parameter (Est. Error)	95% Credible Interval	Probability of Direction (pd)
**Affect arousal**	**Fixed effects**			
	Intercept, γ_00_	–0.89 (0.61)	[–2.07, 0.31]	93.50%
	Day, γ_20_	0.03* (0.01)	[0.02, 0.04]	100%
	Overall air pollution (trait), γ_01_	–0.10 (0.11)	[–0.31, 0.12]	83.30%
	Daily air pollution (state), γ_10_	–0.01* (0.01)	[–0.02, –0.00]	97.83%
	Overall air pollution (trait) * Daily air pollution (state), γ_11_	0.002* (0.00)	[–0.00, 0.00]	97.17%
	**Random effects**			
	Intercept, σ_u0_	7.16* (0.42)	[6.39, 8.04]	100%
	Daily air pollution (state), σ_u1_	0.01* (0.01)	[0.00, 0.04]	100%
	Corr. Intercept, Daily air pollution (state), *r*_u0u1_	–0.07 (0.42)	[–0.87, 0.84]	57.77%
	Residual, σ_e_	8.63* (0.07)	[8.50, 8.77]	100%
	Observations	8,250		
	ICC	0.41		
	Marginal R^2^	0.007		
	Conditional R^2^	0.398		
**Affect valence**	**Fixed effects**			
	Intercept, γ_00_	35.03* (1.84)	[31.27, 38.60]	100%
	Day, γ_20_	0.03* (0.01)	[0.01, 0.06]	99.77%
	Overall air pollution (trait), γ_01_	0.17 (0.30)	[–0.41, 0.77]	72.60%
	Daily air pollution (state), γ_10_	0.02 (0.02)	[–0.01, 0.06]	92.40%
	Overall air pollution (trait) * Daily air pollution (state), γ_11_	–0.005 (0.00)	[–0.01, 0.00]	94.40%
	**Random effects**			
	Intercept, σ_u0_	21.50* (1.25)	[19.09, 24.07]	100%
	Daily air pollution (state), σ_u1_	0.13* (0.02)	[0.09, 0.17]	100%
	Corr. Intercept, Daily air pollution (state), *r*_u0u1_	–0.01 (0.13)	[–0.25, 0.24]	55.97%
	Residual, σ_e_	18.20* (0.15)	[17.91, 18.50]	100%
	Observations	8,250		
	ICC	0.59		
	Marginal R^2^	0.003		
	Conditional R^2^	0.574		

Analysis is based on 8,250 days nested within 150 persons.

* indicates probability of direction (pd) of the parameter > 95%.

### Affect arousal

As seen in the first model in Table 2 and displayed in the top panels of Fig 2, the prototypical individual’s baseline level of *arousal* was γ_00_ = –0.89 (probability of direction, or pd = 93.50%) and increased slightly across days, γ_20_ = 0.03 (pd = 100%). In line with hypotheses, the prototypical individual’s ASAP manifesting in *arousal* was γ_10_ = –0.01 (pd = 97.83%). That is, on days when air pollution is higher than usual by one unit on the 0 to 500 scale, the prototypical individual’s level of *arousal* is –0.01 unit lower on the –100 to 100 scale.

Paralleling the prototypical within-person association captured through articulation of ASAP, there was only weak evidence that individuals with greater overall exposure to air pollution had lower baseline levels of *arousal*, γ_01_ = –0.10 (pd = 83.30%). However, in line with hypotheses, there was stronger evidence that individuals with greater overall exposure to air pollution may have marginally less pronounced ASAP, γ_11_ = 0.002 (pd = 97.17%), suggesting habituation.

Most important for our empirical illustration of the ASAP construct, there were, even after controlling for differences in overall exposure to air pollution, substantial interindividual differences in ASAP, σ_u1_ = 0.01 (pd = 100%), that were not correlated (*r*_u0u1_ = –0.07, pd = 57.77%) with the typically expected differences in individuals’ baseline levels of *arousal*, σ_u0_ = 7.16 (pd = 100%). As displayed in the top left panel of Fig 2, the results confirm that interindividual differences in ASAP (shown by the thin black lines) can be discerned using intensive longitudinal data, and that the prototypical individual’s ASAP manifests in the expected way (negative slope of the thick blue line) for affect *arousal*. In other words, there are significant between-person differences in within-person sensitivity of affect *arousal* to daily changes in air pollution.

### Affect valence

As seen in the second model in Table 2 and displayed in the bottom panels of Fig 2, the prototypical individual’s baseline level of *valence* was γ_00_ = 35.03 (pd = 100%) and increased slightly across days, γ_20_ = 0.03 (pd = 99.77%). However, there was not strong evidence that the prototypical individual’s ASAP manifesting in *valence* was different than zero (γ_10_ = 0.02; pd = 92.40%).

Paralleling the prototypical within-person association captured through articulation of ASAP, there was no evidence that individuals with greater overall exposure to air pollution had lower baseline levels of *valence*, γ_01_ = 0.17 (pd = 72.60%). In line with hypotheses, there was a hint of evidence that individuals with greater overall exposure to air pollution may have less pronounced ASAP, γ_11_ = –0.005 (pd = 94.40%), suggesting habituation.

Most important for our empirical illustration of the ASAP construct, there were, even after controlling for differences in overall exposure to air pollution, substantial interindividual differences in ASAP, σ_u1_ = 0.13 (pd = 100%), that were not correlated (*r*_u0u1_ = –0.01, pd = 55.97%) with the typically expected differences in individuals’ baseline levels of *valence*, σ_u0_ = 21.50 (pd = 100%). As displayed in the bottom left panel of Fig 2, the results confirm that interindividual differences in ASAP (shown by the thin black lines) can be discerned using intensive longitudinal data, even though the prototypical individual’s ASAP is not different than zero (thick blue line) for affect *valence*. In other words, there are significant between-person differences in within-person sensitivity of affect *valence* to daily changes in air pollution.

## Discussion

This paper introduced the ASAP construct and illustrated its measurement using intensive longitudinal data. Our empirical illustration demonstrates the viability of using air pollution data obtained from local air quality monitors and psychological data obtained in experience sampling studies to articulate individuals’ ASAP. As expected, we found that ASAP was indeed discernable, that the prototypical individual’s affect *arousal* was lower than usual on days with higher than usual air pollution, and that—most importantly—there were indeed substantial interindividual differences in ASAP for both affect *arousal* and affect *valence*. Results on how ASAP differed in relation to individuals’ chronic exposure to air pollution suggested habituation effects for both affect *arousal* and affect *valence*, but we maintain a cautious interpretation of that finding for reasons outlined below.

Our findings that individuals’ day-to-day affect may be disrupted by air pollution has three implications. First, ASAP could help partially explain one of the mechanisms by which exposure to air pollution increases longer-term risk for adverse mental health outcomes, like symptoms of anxiety and depression, found in prior research [39–43]. Put differently, climate change is exposing more individuals to air pollution that disturbs affect. Since disturbed affect is implicated in 40–75% of mental disorders [18–21], a more detailed understanding of ASAP can help support climate adaptation by developing strategies to safeguard mental health. Second, since affect is a key driver of individuals’ climate mitigation and adaptation behaviors [24–26], if air pollution blunts an individual’s affect, the blunting might carry over into a (lack of) climate action [27]. Lack of mitigation behaviors could substantially impede the international goal of limiting warming to 1.5 degrees Celsius above pre-industrial levels, as enshrined in the Paris Agreement [66]. Lack of adaptation behaviors could also impede the protective actions taken by individuals exposed to climate hazards. For example, if people’s affect is blunted due to wildfire-induced air pollution during the summer, they may take fewer adaptation behaviors in response to both air pollution and heat waves, with serious implications for their wellbeing. Third, although the whole sample lived in one geographic region and experienced comparable exposures to air pollution, they still presented different ASAP. Aligning with the IPCC’s [7] redefinition of climate vulnerability, these interindividual differences in ASAP underscore the distinction between *sensitivity* and *exposure* to climate change.

### Applications for policy and practice

Climate adaptation is “the process of adjustment to actual or expected climate and its effects, in order to moderate harm or exploit beneficial opportunities” [67]. Leveraging the centrality of affect in psychological processes, ASAP exemplifies the type of constructs that can help us monitor and support mental health in the face of climate hazards, such as climate change-induced air pollution (e.g., wildfire smoke). First, ASAP can be used to inform climate vulnerability assessments. As assessors of climate vulnerability begin looking beyond communities’ *exposure* to climate hazards and toward communities’ *sensitivity* and *adaptive capacity* [1, 3–6, 8, 9], constructs like ASAP could be integrated into climate vulnerability assessments as indicators for sensitivity to climate change. It is important to note, however, that because vulnerability is a socially produced state where people are made vulnerable to climate change through systems of power, ASAP should be considered one component of a much larger system. Second, ASAP can guide policy and investment decisions by highlighting differences in sensitivity to air pollution across individuals, when the extent of these differences may otherwise be obscured in population-level data. For example, while unexamined in this study, it is possible that certain populations, such as older adults or children, exhibit higher ASAP than others. While measures of ASAP do not address the root causes of air pollution, or its inequitable distribution [33], they can help quantify the effects of air pollution where it is already being felt and direct resources accordingly. Third, ASAP can enable the design of personalized interventions that support affect in the face of air pollution exposure. For instance, the air pollution threshold at which someone receives an air quality alert and the protective actions recommended to them could be personalized based on their ASAP. Through each of these pathways, ASAP provides opportunity to better integrate mental health in climate adaptation policies, plans, and programs.

### Limitations and outlook

As ASAP-related studies develop, it is useful to consider several limitations of our analysis. First, our data featured relatively low overall levels of day-to-day variability in air pollution. Greater variance of air pollution might uncover stronger effects and larger differences in individuals’ ASAP. Second, air quality data for the five major pollutants was not consistently collected by the EPA during the iSAHIB study period. Our examination of missingness patterns provided no indication that the EPA’s air quality monitors worked selectively for particular pollutants on particular days. However, missingness in the pollutant data means that AQI values may be underestimated. Third, we used spatially monadic, county-level AQI data, which assumes that sensors at a single location characterize environmental exposures over a larger area. Since many people are mobile during the day and air pollution exposures vary over space and time [68, 69], air pollution data that is more specific to participants’ homes or mobility paths would enable more precise measurement of ASAP. Fourth, although age heterogeneous, the sample was racially, ethnically, and geographically homogeneous. In addition, iSAHIB data was collected in 2010 and 2011. Our results thus may not reflect the ASAP of more diverse populations today. Fifth, and importantly for interpretation of habituation findings, our analysis did not address the possibility that individuals may already be taking actions to protect themselves from air pollution, such as reducing outdoor exercise on more polluted days, and thus may underestimate individuals’ actual ASAP.

Future research on ASAP should consider incorporating more study sites to widen the range of AQI values, obtaining more granular measures of air pollution, sampling more diverse individuals, and collecting data about any protective actions that individuals are already taking to cope with air pollution. Moreover, future work can apply the methodology proposed here to expand the measurement of sensitivity to climate change (affective or otherwise) to other climate hazards, such as flooding or drought.

## Conclusion

In this paper, we proposed a new intraindividual variability construct, *affective sensitivity to air pollution* (ASAP), that describes the extent to which an individual’s affective states fluctuate in accordance with daily changes in air pollution. Our empirical illustration found that there were indeed substantial interindividual differences in ASAP, suggesting that repeated measures of individuals’ day-to-day affect provide a new way of measuring sensitivity to climate change. As climate change threatens human health and wellbeing worldwide, ASAP contributes to discourse around the conceptualization and measurement of individuals’ climate vulnerability. This new construct can be leveraged to better integrate affect and mental health in climate adaptation policies, plans, and programs.

## Supporting information

S1 Table Sensitivity analyses examining subsets of emotions. (DOCX)

S1 Dataset Data analyzed in this study. (CSV)

## References

[1] Adger WN. Vulnerability. Global environmental change. 2006 Aug 1;16(3):268–81.

[2] Füssel HM, Klein RJ. Climate change vulnerability assessments: an evolution of conceptual thinking. Climatic change. 2006 Apr;75(3):301–29.

[3] Sharma J, Ravindranath NH. Applying IPCC 2014 framework for hazard-specific vulnerability assessment under climate change. Environmental Research Communications. 2019 Jun 1;1(5):051004.

[4] IPCC, 2007: Summary for Policymakers. In: Climate Change 2007: *Impacts*, *Adaptation and Vulnerability*. Contribution of Working Group II to the Fourth Assessment Report of the Intergovernmental Panel on Climate Change [M.L. Parry, O.F. Canziani, J.P. Palutikof, P.J. van der Linden and C.E. Hanson (eds.)], Cambridge University Press, Cambridge, UK, 7–22.

[5] Smit B, Wandel J. Adaptation, adaptive capacity and vulnerability. Global environmental change. 2006 Aug 1;16(3):282–92.

[6] IPCC, 2014: Summary for policymakers. In: *Climate Change 2014*: *Impacts*, *Adaptation*, *and Vulnerability*. Contribution of Working Group II to the Fifth Assessment Report of the Intergovernmental Panel on Climate Change [Field, C.B., V.R. Barros, D.J. Dokken, K.J. Mach, M.D. Mastrandrea, T.E. Bilir, M. Chatterjee, K.L. Ebi, Y.O. Estrada, R.C. Genova, B. Girma, E.S. Kissel, A.N. Levy, S. MacCracken, P.R. Mastrandrea, and L.L. White (eds.)]. Cambridge University Press, Cambridge, United Kingdom and New York, NY, USA, pp. 1–32.

[7] IPCC, 2022: Summary for Policymakers. In: *Climate Change 2022*: *Impacts*, *Adaptation*, *and Vulnerability*. Contribution of Working Group II to the Sixth Assessment Report of the Intergovernmental Panel on Climate Change [H.-O. Pörtner, D.C. Roberts, M. Tignor, E.S. Poloczanska, K. Mintenbeck, A. Alegría, M. Craig, S. Langsdorf, S. Löschke, V. Möller, A. Okem, B. Rama (eds.)]. Cambridge University Press, Cambridge, UK and New York, NY, USA, pp. 3–33.

[8] Eriksen SH, Nightingale AJ, Eakin H. Reframing adaptation: The political nature of climate change adaptation. Global Environmental Change. 2015 Nov 1;35:523–33.

[9] Kuhlicke C, de Brito MM, Bartkowski B, Botzen W, Doğulu C, Han S, et al. Spinning in circles? A systematic review on the role of theory in social vulnerability, resilience and adaptation research. Global Environmental Change. 2023 May 1;80:102672.

[10] Gross JJ. Emotion regulation: Conceptual and empirical foundations. Handbook of emotion regulation. 2014;2:3–20.

[11] Mroczek DK, Almeida DM. The effect of daily stress, personality, and age on daily negative affect. Journal of personality. 2004 Apr;72(2):355–78. doi: 10.1111/j.0022-3506.2004.00265.x 15016068

[12] Gross JJ, Sutton SK, Ketelaar T. Relations between affect and personality: Support for the affect-level and affective-reactivity views. Personality and social psychology bulletin. 1998 Mar;24(3):279–88.

[13] Bolger N, Schilling EA. Personality and the problems of everyday life: The role of neuroticism in exposure and reactivity to daily stressors. Journal of personality. 1991 Sep;59(3):355–86. doi: 10.1111/j.1467-6494.1991.tb00253.x 1960637

[14] Miller DJ, Vachon DD, Lynam DR. Neuroticism, negative affect, and negative affect instability: Establishing convergent and discriminant validity using ecological momentary assessment. Personality and individual differences. 2009 Dec 1;47(8):873–7. doi: 10.1016/j.paid.2009.07.007 20160976 PMC2760940

[15] Leger KA, Turiano NA, Bowling W, Burris JL, Almeida DM. Personality traits predict long-term physical health via affect reactivity to daily stressors. Psychological science. 2021 May;32(5):755–65. doi: 10.1177/0956797620980738 33882261 PMC8258308

[16] Carstensen LL, Turan B, Scheibe S, Ram N, Ersner-Hershfield H, Samanez-Larkin GR, et al. Emotional experience improves with age: evidence based on over 10 years of experience sampling. Psychology and aging. 2011 Mar;26(1):21. doi: 10.1037/a0021285 20973600 PMC3332527

[17] Schöllgen I, Morack J, Infurna FJ, Ram N, Gerstorf D. Health sensitivity: Age differences in the within-person coupling of individuals’ physical health and well-being. Developmental Psychology. 2016 Nov;52(11):1944. doi: 10.1037/dev0000171 27786533 PMC5096387

[18] Kring AM. Emotion disturbances as transdiagnostic processes in psychopathology. Handbook of emotion. 2008;3:691–705.

[19] Jazaieri H, Urry HL, Gross JJ. Affective disturbance and psychopathology: An emotion regulation perspective. Journal of Experimental Psychopathology. 2013 Dec;4(5):584–99.

[20] Gross JJ, Jazaieri H. Emotion, emotion regulation, and psychopathology: An affective science perspective. Clinical psychological science. 2014 Jul;2(4):387–401.

[21] Gross JJ, Uusberg H, Uusberg A. Mental illness and well-being: an affect regulation perspective. World Psychiatry. 2019 Jun;18(2):130–9. doi: 10.1002/wps.20618 31059626 PMC6502417

[22] Witte K. Putting the fear back into fear appeals: The extended parallel process model. Communications Monographs. 1992 Dec 1;59(4):329–49.

[23] Van der Linden S. The social-psychological determinants of climate change risk perceptions: Towards a comprehensive model. Journal of Environmental Psychology. 2015 Mar 1;41:112–24.

[24] Xie B, Brewer MB, Hayes BK, McDonald RI, Newell BR. Predicting climate change risk perception and willingness to act. Journal of Environmental Psychology. 2019 Oct 1;65:101331.

[25] van Valkengoed AM, Steg L. Meta-analyses of factors motivating climate change adaptation behaviour. Nature climate change. 2019 Feb;9(2):158–63.

[26] Brosch T. Affect and emotions as drivers of climate change perception and action: a review. Current Opinion in Behavioral Sciences. 2021 Dec 1;42:15–21.

[27] Bickel LA, Preston SD. Environmental impassivity: Blunted emotionality undermines concern for the environment. Emotion. 2023 Jun;23(4):1175. doi: 10.1037/emo0001072 35925709

[28] Stathopoulou E, Mihalakakou G, Santamouris M, Bagiorgas HS. On the impact of temperature on tropospheric ozone concentration levels in urban environments. Journal of Earth System Science. 2008 Jun;117:227–36.

[29] Kinney PL. Interactions of climate change, air pollution, and human health. Current environmental health reports. 2018 Mar;5:179–86. doi: 10.1007/s40572-018-0188-x 29417451

[30] Campbell-Lendrum D, Prüss-Ustün A. Climate change, air pollution and noncommunicable diseases. Bulletin of the World Health Organization. 2019 Feb 2;97(2):160. doi: 10.2471/BLT.18.224295 30728622 PMC6357572

[31] Burke M, Driscoll A, Heft-Neal S, Xue J, Burney J, Wara M. The changing risk and burden of wildfire in the United States. Proceedings of the National Academy of Sciences. 2021 Jan 12;118(2):e2011048118. doi: 10.1073/pnas.2011048118 33431571 PMC7812759

[32] World Health Organization. Ambient air pollution: A global assessment of exposure and burden of disease. 2016.

[33] Grineski S, Bolin B, Boone C. Criteria air pollution and marginalized populations: environmental inequity in metropolitan Phoenix, Arizona. Social Science Quarterly. 2007 Jun;88(2):535–54.

[34] Landrigan PJ, Fuller R, Acosta NJ, Adeyi O, Arnold R, Baldé AB, et al. The Lancet Commission on pollution and health. The lancet. 2018 Feb 3;391(10119):462–512. 10.1016/S0140-6736(17)32345-0 29056410

[35] Kampa M, Castanas E. Human health effects of air pollution. Environmental pollution. 2008 Jan 1;151(2):362–7. doi: 10.1016/j.envpol.2007.06.012 17646040

[36] Franklin BA, Brook R, Pope CA III. Air pollution and cardiovascular disease. Current problems in cardiology. 2015 May 1;40(5):207–38. doi: 10.1016/j.cpcardiol.2015.01.003 25882781

[37] Dockery DW, Pope CA. Acute respiratory effects of particulate air pollution. Annual review of public health. 1994 May;15(1):107–32. doi: 10.1146/annurev.pu.15.050194.000543 8054077

[38] Turner MC, Andersen ZJ, Baccarelli A, Diver WR, Gapstur SM, Pope CA III, et al. Outdoor air pollution and cancer: An overview of the current evidence and public health recommendations. CA: a cancer journal for clinicians. 2020 Nov;70(6):460–79. doi: 10.3322/caac.21632 32964460 PMC7904962

[39] Mehta AJ, Kubzansky LD, Coull BA, Kloog I, Koutrakis P, Sparrow D, et al. Associations between air pollution and perceived stress: the Veterans Administration Normative Aging Study. Environmental Health. 2015 Dec;14(1):1–0. doi: 10.1186/1476-069X-14-10 25627872 PMC4417295

[40] Miller JG, Gillette JS, Kircanski K, LeMoult J, Gotlib IH. Air pollution is associated with elevated HPA-Axis response to stress in anxious adolescent girls. Comprehensive Psychoneuroendocrinology. 2020 Nov 1;4:100015. doi: 10.1016/j.cpnec.2020.100015 35755623 PMC9216601

[41] Pun VC, Manjourides J, Suh H. Association of ambient air pollution with depressive and anxiety symptoms in older adults: results from the NSHAP study. Environmental health perspectives. 2017 Mar;125(3):342–8. doi: 10.1289/EHP494 27517877 PMC5332196

[42] Szyszkowicz M, Zemek R, Colman I, Gardner W, Kousha T, Smith-Doiron M. Air pollution and emergency department visits for mental disorders among youth. International journal of environmental research and public health. 2020 Jun;17(12):4190. doi: 10.3390/ijerph17124190 32545456 PMC7345689

[43] Reuben A, Arseneault L, Beddows A, Beevers SD, Moffitt TE, Ambler A, et al. Association of air pollution exposure in childhood and adolescence with psychopathology at the transition to adulthood. JAMA Network Open. 2021 Apr 1;4(4):e217508-. doi: 10.1001/jamanetworkopen.2021.7508 33909054 PMC8082321

[44] Zanobetti A, Redline S, Schwartz J, Rosen D, Patel S, O’Connor GT, et al. Associations of PM10 with sleep and sleep-disordered breathing in adults from seven US urban areas. American journal of respiratory and critical care medicine. 2010 Sep 15;182(6):819–25. 20508218 10.1164/rccm.200912-1797OC PMC2949406

[45] Yu H, Yu M, Gordon SP, Zhang R. The association between ambient fine particulate air pollution and physical activity: a cohort study of university students living in Beijing. International Journal of Behavioral Nutrition and Physical Activity. 2017 Dec;14(1):1–0. 28982357 10.1186/s12966-017-0592-x PMC5629773

[46] An R, Zhang S, Ji M, Guan C. Impact of ambient air pollution on physical activity among adults: a systematic review and meta-analysis. Perspectives in public health. 2018 Mar;138(2):111–21. doi: 10.1177/1757913917726567 28829249

[47] Shen YL, Liu WT, Lee KY, Chuang HC, Chen HW, Chuang KJ. Association of PM2. 5 with sleep-disordered breathing from a population-based study in Northern Taiwan urban areas. Environmental Pollution. 2018 Feb 1;233:109–13. doi: 10.1016/j.envpol.2017.10.052 29059625

[48] Rosenthal DG, Vittinghoff E, Tison GH, Pletcher MJ, Olgin JE, Grandis DJ, et al. Assessment of accelerometer-based physical activity during the 2017–2018 California wildfire seasons. JAMA Network Open. 2020 Sep 1;3(9):e2018116-. doi: 10.1001/jamanetworkopen.2020.18116 32997120 PMC7527871

[49] Doubleday A, Choe Y, Isaksen TM, Errett NA. Urban bike and pedestrian activity impacts from wildfire smoke events in Seattle, WA. Journal of Transport & Health. 2021 Jun 1;21:101033.

[50] Chevance G, Fresán U, Hekler E, Edmondson D, Lloyd SJ, Ballester J, et al. Thinking health-related behaviors in a climate change context: a narrative review. Annals of Behavioral Medicine. 2023 Mar 1;57(3):193–204. doi: 10.1093/abm/kaac039 35861123 PMC10074036

[51] Potter S, Röcke C, Gerstorf D, Brose A, Kolodziejczak K, Hoppmann CA, et al. Partner pain and affect in the daily lives of older couples. The Journals of Gerontology: Series B. 2022 Jul 1;77(7):1197–209. doi: 10.1093/geronb/gbab188 34653253

[52] Potter S, Gerstorf D, Schmiedek F, Drewelies J, Wolff JK, Brose A. Health sensitivity in the daily lives of younger and older adults: Correlates and longer-term change in health. Aging & Mental Health. 2022 May 19;26(6):1261–9. doi: 10.1080/13607863.2021.1913475 33938784

[53] Ram N, Conroy DE, Pincus AL, Lorek A, Rebar A, Roche MJ, et al. Examining the interplay of processes across multiple time-scales: Illustration with the Intraindividual Study of Affect, Health, and Interpersonal Behavior (iSAHIB). Research in Human Development. 2014 Apr 3;11(2):142–60. doi: 10.1080/15427609.2014.906739 26989350 PMC4792298

[54] United States Environmental Protection Agency (EPA). Air Data. https://www.epa.gov/outdoor-air-quality-data. Accessed April 2022.

[55] Maher JP, Pincus AL, Ram N, Conroy DE. Daily physical activity and life satisfaction across adulthood. Developmental psychology. 2015 Oct;51(10):1407. doi: 10.1037/dev0000037 26280838 PMC4579061

[56] Lydon DM, Ram N, Conroy DE, Pincus AL, Geier CF, Maggs JL. The within-person association between alcohol use and sleep duration and quality in situ: An experience sampling study. Addictive behaviors. 2016 Oct 1;61:68–73. doi: 10.1016/j.addbeh.2016.05.018 27249804 PMC4915974

[57] Ram N, Benson L, Brick TR, Conroy DE, Pincus AL. Behavioral landscapes and earth mover’s distance: A new approach for studying individual differences in density distributions. Journal of Research in Personality. 2017 Aug 1;69:191–205. doi: 10.1016/j.jrp.2016.06.010 28959082 PMC5612642

[58] Vogel N, Ram N, Conroy DE, Pincus AL, Gerstorf D. How the social ecology and social situation shape individuals’ affect valence and arousal. Emotion. 2017 Apr;17(3):509. doi: 10.1037/emo0000244 27869467

[59] AirNow. Air Quality Index (AQI) Basics. https://www.airnow.gov/aqi/aqi-basics/. Accessed April 2023.

[60] Russell JA. A circumplex model of affect. Journal of personality and social psychology. 1980 Dec;39(6):1161.

[61] Kuppens P, Van Mechelen I, Nezlek JB, Dossche D, Timmermans T. Individual differences in core affect variability and their relationship to personality and psychological adjustment. Emotion. 2007 May;7(2):262. doi: 10.1037/1528-3542.7.2.262 17516805

[62] Snijders TA, Bosker R. Multilevel analysis: an introduction to basic and advanced multilevel modeling. 1999 (pp. 266–266).

[63] Bolger N, Laurenceau JP. Intensive longitudinal methods: An introduction to diary and experience sampling research. Guilford press; 2013 Feb 14.

[64] Röcke C, Brose A. Intraindividual variability and stability of affect and well-being. GeroPsych. 2013 Aug 23.

[65] Bürkner PC. brms: An R package for Bayesian multilevel models using Stan. Journal of statistical software. 2017 Aug 29;80:1–28.

[66] United Nations. Paris Agreement. 2015.

[67] IPCC, 2018: *Annex I*: *Glossary*. *In*: *Global Warming of 1*.*5°C*. *An IPCC Special Report on the impacts of global warming of 1*.*5°C above pre-industrial levels and related global greenhouse gas emission pathways*, *in the context of strengthening the global response to the threat of climate change*, *sustainable development*, *and efforts to eradicate poverty* [Masson-Delmotte, V., P. Zhai, H.-O. Pörtner, D. Roberts, J. Skea, P.R. Shukla, A. Pirani, W. Moufouma-Okia, C. Péan, R. Pidcock, S. Connors, J.B.R. Matthews, Y. Chen, X. Zhou, M.I. Gomis, E. Lonnoy, T. Maycock, M. Tignor, and T. Waterfield (eds.)]. Cambridge University Press, Cambridge, UK and New York, NY, USA, pp. 541–562.

[68] Matthews SA, Yang TC. Spatial polygamy and contextual exposures (spaces) promoting activity space approaches in research on place and health. American behavioral scientist. 2013 Aug;57(8):1057–81. doi: 10.1177/0002764213487345 24707055 PMC3975622

[69] Schneider CM, Belik V, Couronné T, Smoreda Z, González MC. Unravelling daily human mobility motifs. Journal of The Royal Society Interface. 2013 Jul 6;10(84):20130246. doi: 10.1098/rsif.2013.0246 23658117 PMC3673164

