# Supplementary material for: Affective Sensitivity to Air Pollution (ASAP): Person-specific associations between daily air pollution and affective states
Source: PLoS One. 2024 Aug 7;19(8):e0307430. doi: 10.1371/journal.pone.0307430 (PMC11305556; doi:10.1371/journal.pone.0307430)
Supplement: S1 Table — (DOCX) [file pone.0307430.s001.docx]

**Table S1. Sensitivity analyses examining subsets of emotions.**

|  |  | Parameter (Est. Error) | 95% Credible Interval | Probability of Direction (pd) |  |
| --- | --- | --- | --- | --- | --- |
| **Positive high arousal *(alert, enthusiastic, excited, happy, proud)*** | **Fixed effects** |  |  |  |  |
|  | Intercept, γ_00_ | 55.91* (1.25) | [53.64, 58.37] | 100% |  |
|  | Day, γ_20_ | 0.00 (0.01) | [–0.01, 0.02] | 76.03% |  |
|  | Average air pollution (trait), γ_01_ | –0.03 (0.22) | [–0.47, 0.40] | 54.77% |  |
|  | Daily air pollution (state), γ_10_ | 0.01 (0.01) | [–0.01, 0.03] | 80.37% |  |
|  | Average air pollution (trait) * Daily air pollution (state), γ_11_ | –0.00 (0.00) | [–0.01, 0.00] | 86.67% |  |
|  | **Random effects** |  |  |  |  |
|  | Intercept, σ_u0_ | 15.64* (0.94) | [13.74, 17.58] | 100% |  |
|  | Daily air pollution (state), σ_u1_ | 0.07* (0.01) | [0.05, 0.09] | 100% |  |
|  | Corr. Intercept, Daily air pollution (state), *r*_u0u1_ | –0.19 (0.14) | [–0.46, 0.07] | 91.47% |  |
|  | Residual, σ_e_ | 11.12 (0.09) | [10.94, 11.30] | 100% |  |
|  | Observations | 8,250 |  |  |  |
|  | ICC | 0.67 |  |  |  |
|  | Marginal R^2^ | 0.002 |  |  |  |
|  | Conditional R^2^ | 0.660 |  |  |  |
| **Positive low arousal *(calm, content, peaceful, relaxed, satisfied)*** | | **Fixed effects** |  |  |  |
|  |  | Intercept, γ_00_ | 58.83* (1.20) | [56.39, 61.21] | 100% |
|  |  | Day, γ_20_ | –0.02* (0.01) | [–0.03, –0.00] | 99.43% |
|  |  | Average air pollution (trait), γ_01_ | 0.03 (0.22) | [–0.44, 0.43] | 56.97% |
|  |  | Daily air pollution (state), γ_10_ | 0.02* (0.01) | [–0.00, 0.04] | 95.27% |
|  |  | Average air pollution (trait) * Daily air pollution (state), γ_11_ | –0.00 (0.00) | [–0.01, 0.00] | 91.67% |
|  | | **Random effects** |  |  |  |
|  | | Intercept, σ_u0_ | 14.93* (0.92) | [13.18, 16.77] | 100% |
|  | | Daily air pollution (state), σ_u1_ | 0.09* (0.01) | [0.07, 0.12] | 100% |
|  | | Corr. Intercept, Daily air pollution (state), *r*_u0u1_ | –0.03 (0.12) | [–0.26, 0.21] | 58.17% |
|  | | Residual, σ_e_ | 12.39* (0.10) | [12.20, 12.58] | 100% |
|  | | Observations | 8,250 |  |  |
|  | | ICC | 0.60 |  |  |
|  | | Marginal R^2^ | 0.003 |  |  |
|  | | Conditional R^2^ | 0.586 |  |  |
| **Negative high arousal *(embarrassed, nervous, stressed, tense, upset)*** | | **Fixed effects** |  |  |  |
|  |  | Intercept, γ_00_ | 22.70* (0.90) | [21.00, 24.44] | 100% |
|  |  | Day, γ_20_ | –0.02* (0.01) | [–0.03, –0.00] | 98.97% |
|  |  | Average air pollution (trait), γ_01_ | –0.27 (0.18) | [–0.62, 0.09] | 92.57% |
|  |  | Daily air pollution (state), γ_10_ | –0.02* (0.01) | [–0.04, 0.00] | 95.60% |
|  |  | Average air pollution (trait) * Daily air pollution (state), γ_11_ | 0.00* (0.00) | [0.00, 0.01] | 98.33% |
|  | | **Random effects** |  |  |  |
|  | | Intercept, σ_u0_ | 11.50* (0.72) | [10.22, 13.05] | 100% |
|  | | Daily air pollution (state), σ_u1_ | 0.05* (0.02) | [0.01, 0.08] | 100% |
|  | | Corr. Intercept, Daily air pollution (state), *r*_u0u1_ | –0.11 (0.24) | [–0.64, 0.33] | 72.57% |
|  | | Residual, σ_e_ | 12.48* (0.10) | [12.30, 12.68] | 100% |
|  | | Observations | 8,250 |  |  |
|  | | ICC | 0.46 |  |  |
|  | | Marginal R^2^ | 0.007 |  |  |
|  | | Conditional R^2^ | 0.450 |  |  |
| **Negative low arousal *(bored, depressed, disappointed, sad, sluggish)*** | | **Fixed effects** |  |  |  |
|  |  | Intercept, γ_00_ | 21.78* (0.93) | [20.05, 23.69] | 100% |
|  |  | Day, γ_20_ | –0.06* (0.01) | [–0.08, –0.05] | 100% |
|  |  | Average air pollution (trait), γ_01_ | –0.09 (0.17) | [–0.41, 0.24] | 71.17% |
|  |  | Daily air pollution (state), γ_10_ | –0.00 (0.01) | [–0.02, 0.01] | 66.67% |
|  |  | Average air pollution (trait) * Daily air pollution (state), γ_11_ | 0.00 (0.00) | [–0.00, 0.00] | 74.97% |
|  | | **Random effects** |  |  |  |
|  | | Intercept, σ_u0_ | 10.61* (0.63) | [9.47, 11.94] | 100% |
|  | | Daily air pollution (state), σ_u1_ | 0.06* (0.01) | [0.04, 0.08] | 100% |
|  | | Corr. Intercept, Daily air pollution (state), *r*_u0u1_ | –0.11 (0.15) | [–0.41, 0.18] | 77.87% |
|  | | Residual, σ_e_ | 10.63* (0.09) | [10.46, 10.81] | 100% |
|  | | Observations | 8,250 |  |  |
|  | | ICC | 0.50 |  |  |
|  | | Marginal R^2^ | 0.008 |  |  |
|  | | Conditional R^2^ | 0.487 |  |  |

Analysis based on 8,250 days nested within 150 persons.

* indicates probability of direction (pd) of the parameter > 95%.
